# Does ovarian stimulation accelerate the progression of BI-RADS categories? a propensity score matching-based retrospective cohort study

**DOI:** 10.3389/fendo.2026.1844265

**Published:** 2026-08-11

**Authors:** Tingting Wang, Jinxin Ren, Zhaokang Qi, Shuai Zhao, Yi Yu, Fang Lian, Hao Wang

**Affiliations:** 1 The First Clinical Medical College, Shandong University of Traditional Chinese Medicine, Jinan, China; 2 Department of Reproduction and Genetics, Affiliated Hospital of Shandong University of Traditional Chinese Medicine, Jinan, China

**Keywords:** assisted reproductive technology, BI-RADS, breast cancer risk, lactation, ovarian stimulation, propensity score matching

## Abstract

**Purpose:**

To investigate whether controlled ovarian stimulation (COS), as part of assisted reproductive technology (ART), accelerates the progression of BI-RADS categories.

**Methods:**

This was a single-center, retrospective, propensity score matching (PSM) cohort study conducted from January 2020 to January 2025. A total of 9, 632 women were initially enrolled. After 1:1 PSM, the final analyzed cohort comprised 229 pairs of women aged <35 years (younger group) and 154 pairs of women aged ≥35 years (older group). The exposed group comprised women who underwent COS for IVF/ICSI treatment, while the control group consisted of women who underwent routine physical examinations during the same period with no history of ART treatment. The primary outcome was the change (escalation or regression) in BI-RADS category from baseline to follow-up (≥12 months), blindly re-evaluated by two senior sonographers. Propensity score matching was performed to balance baseline characteristics between groups, and subgroup and trend analyses were conducted.

**Results:**

After PSM, baseline characteristics were well balanced between the groups. In the <35 years cohort, the exposed group showed significantly higher rates of both BI-RADS category escalation (30.13% vs. 13.54%, P = 0.019) and regression (14.84% vs. 6.98%, P = 0.007) compared with the control group. In the ≥35 years cohort, the escalation rate was significantly higher in the exposed group than in the control group (36.36% vs. 12.33%, P<0.001), whereas no significant difference was observed in regression rates. Trend analysis revealed that among women aged ≥35 years, the risk of BI-RADS category escalation increased significantly with the number of stimulation cycles (trend P = 0.04). All 12 patients who underwent biopsy due to escalation to BI-RADS 4A had benign pathological findings. Regarding pregnancy outcomes, the <35 years group demonstrated significantly higher clinical pregnancy and live birth rates, as well as a lower miscarriage rate, compared with the ≥35 years group.

**Conclusion:**

COS is associated with an increased short-term risk of BI-RADS category progression, particularly among women aged ≥35 years. However, it is crucial to note that all 12 patients who underwent biopsy following an upgrade to BI-RADS 4A had benign pathological findings. This suggests that the observed imaging changes may represent a transient, hormone-driven breast tissue response rather than an indicator of malignant transformation. The higher regression rate observed in younger women in the exposed group may be related to subsequent pregnancy and lactation, but this remains speculative. These findings highlight the importance of age-stratified imaging surveillance during ART, rather than implying an elevated risk of breast cancer. Long-term prospective studies are warranted to validate these observations.

## 1 Introduction

Breast cancer ranks as the most common malignancy among women in the Asia-Pacific region, with a continuously increasing disease burden (1). Concurrently, the incidence among young women aged <50 years in this region is exhibiting a rapid upward trend, rendering the prevention and management of breast cancer in women of reproductive age a critical public health priority (2, 3).

In Asia, particularly in China, many women of reproductive age face difficulties in natural conception due to reproductive system disorders, such as polycystic ovary syndrome, endometriosis, diminished ovarian reserve, or tubal pathology, or delayed childbearing influenced by social and occupational factors. Assisted reproductive technology (ART) has emerged as a critical pathway for fulfilling fertility aspirations, with controlled ovarian stimulation (COS) constituting one of its core components. This process involves the administration of exogenous follicle-stimulating hormone (FSH) to override the monofollicular dominance characteristic of natural cycles, thereby promoting the synchronous development and maturation of multiple follicles, ultimately aiming to obtain an adequate number of oocytes to lay the foundation for subsequent embryo formation (4, 5).

Estrogen is a well-established influential factor in the initiation and progression of breast cancer. Endogenous estrogen participates in the proliferation and differentiation of breast tissue through binding to the estrogen receptor (6, 7), and prolonged or abnormally elevated levels of endogenous estrogen have been demonstrated to increase the risk of breast cancer (8). During ART, the COS process exposes women to a short-term, supraphysiological surge of exogenous estrogen, which may further influence the subsequent risk of breast cancer (9).

Breast Imaging Reporting and Data System (BI-RADS) is an essential imaging tool for assessing breast lesion risk, providing a standardized classification system for guiding clinical decision-making, and holds significant value for young and high-risk populations (10–12). Studies have demonstrated that endogenous or exogenous hormonal stimulation may dynamically influence imaging characteristics such as breast density, fibroglandular tissue, and background parenchymal enhancement (13), with mechanisms potentially involving hormone receptor-mediated responses in breast tissue (14).

Although existing meta-analyses have not definitively established that ART directly increases the risk of breast cancer, several studies suggest that age may serve as an important modulating factor. Some studies have indicated that older women (≥40 years) undergoing ART may have an elevated risk of breast cancer (15); other research has found that increasing age at treatment initiation is associated with a statistically significant, albeit modest, increasing trend in breast cancer risk (16, 17). For instance, a 2018 study including 225, 786 women undergoing ART in the UK demonstrated no overall increased risk of breast cancer, but observed a moderate increasing trend in risk with advancing age at treatment initiation (18).

Currently, whether COS affects the progression of BI-RADS classification remains unclear. Existing evidence is predominantly focused on epidemiological associations between ART and the risk of breast cancer, or confined to short-term effects of hormones on single imaging parameters such as breast density.Therefore, the aim of this study was to investigate, through a retrospective cohort design, whether ovarian stimulation is associated with significant short-term changes in BI-RADS categories in women undergoing routine health screenings, and to analyze associated influencing factors. This study focuses on characterizing the imaging dynamics of breast tissue in response to COS, providing a reference for imaging-based risk monitoring and personalized surveillance strategies in clinical ART practice, rather than establishing a direct link to breast cancer incidence.

## 2 Materials and methods

This was a single-center, retrospective matched cohort study based on an Asian population. All data were collected from the medical record system of the Affiliated Hospital of Shandong University of Traditional Chinese Medicine and linked using unique personal identification numbers to identify the study population, exposure, and outcomes.

### 2.1 Patients

The study population was derived from the Hospital Information System of the Affiliated Hospital of Shandong University of Traditional Chinese Medicine. All women aged 21–50 years who met the eligibility criteria were consecutively enrolled between January 1, 2020, and January 1, 2025. From a reproductive and genetic perspective, women aged ≥35 years were generally defined as of advanced maternal age (19, 20), while those aged <35 years were defined as young women. This classification is also consistent with the population definition in the POSEIDON criteria (21).

The exposed group comprised women aged 21–50 years who underwent at least one cycle of *in vitro* fertilization/intracytoplasmic sperm injection (IVF/ICSI) treatment with COS at the Reproductive Medicine Center.

The control group consisted of women aged 21–50 years who underwent routine health examinations at the Health Management Center during the same period and had no history of ART treatment.

### 2.2 Inclusion criteria

- Baseline breast ultrasound examination (performed within 6 months prior to ovarian stimulation for the exposed group, or at the initial health examination for the control group) classified as BI-RADS 1, 2, or 3;
- At least one complete follow-up breast ultrasound record (≥12 months after the baseline examination);
- Complete baseline data, including age and body mass index (BMI).

### 2.3 Exclusion criteria (any of the following):

- A history of breast cancer or any other malignancy;
- Baseline breast ultrasound findings classified as BI-RADS 4 or 5;
- Missing records of ovarian stimulation (for the exposed grop) or consecutive ultrasound examinations (for the control group).

### 2.4 Exposure

Exposure was defined as COS using exogenous gonadotropins (e.g., FSH, HMG) and/or oral ovulation induction agents (e.g., letrozole, clomiphene citrate) for the purpose of assisted reproduction.

Propensity score matching (PSM) was performed to match the exposed group with the control group at a 1:1 ratio according to the following variables: age (± 3 years), BMI (± 2 kg/m²), gravidity, parity, and baseline BI-RADS classification. After matching, the final analysis cohort was formed.

### 2.5 Non-exposure

Non-exposure (control group) was defined as the absence of any form of ovarian stimulation or ovulation induction therapy during the study observation period. Individuals in the control group were screened from the health examination population and paired with exposed individuals according to the aforementioned matching criteria to ensure comparability between the two groups in terms of key baseline characteristics.

### 2.6 Outcomes

#### 2.6.1 Primary outcome

The primary outcome was the change in BI-RADS classification. All breast ultrasound images were independently and blindly re-reviewed by two attending physicians, each with over five years of experience in breast ultrasound diagnosis, according to the American College of Radiology BI-RADS (5th edition). Both physicians were blinded to the patients’ group allocation and clinical information. Any discrepancies in interpretation were adjudicated by a third senior sonographer with >10 years of experience, and the final decision was used for analysis. Inter-observer agreement was assessed by calculating the weighted kappa coefficient (κw=0.76) for BI-RADS categories (1–4A) on a randomly selected 30% subsample of images.

Definitions: Upgrade was defined as an increase in BI-RADS classification of a lesion in the same breast region on follow-up ultrasound compared with baseline (e.g., from category 2 to 3). Downgrade was defined as a decrease in BI-RADS classification of a lesion in the same breast region on follow-up ultrasound compared with baseline.

Time calculation: Baseline was defined as the date of the most recent ultrasound examination prior to the start of the first ovarian stimulation cycle (for the exposed group) or the date of the initial enrollment ultrasound (for the control group).

The follow-up time point was set at ≥12 months after baseline. Follow-up ultrasound examinations were performed at this time point for both groups, and changes in BI-RADS classification from baseline were recorded at this time point.

Exploratory outcome: For patients who underwent biopsy due to an upgrade to BI-RADS 4A or higher, the pathological diagnosis was recorded.

Scoring: For the purpose of analysis, the following numerical scores were assigned: no significant risk, category 1, 2, 3, and 4a were categorized as 0, 1, 2, and 3 points, respectively.

#### 2.6.2 Secondary outcomes

- The association and trend between the number of stimulation cycles and the risk of BI-RADS upgrade in different age subgroups. Patients in the exposed group were stratified into subgroups according to the number of ovarian stimulation cycles: 1 cycle, 2 cycles, and ≥3 cycles, with 1 cycle used as the reference. Upgrade/downgrade ratio were calculated as the probability of upgrade divided by the probability of downgrade, i.e., upgrade/downgrade ratio = number of patients with BI-RADS upgrade/number of patients with BI-RADS downgrade.
- Pregnancy outcomes in the exposed group
  - Total clinical pregnancy rate (CPR): The overall ratio of transfer cycles to clinical pregnancies.
  - Total CPR: Number of clinical pregnancies/total number of embryo transfer cycles × 100%.
  - Total live birth rate (LBR): The proportion of cycles achieving at least one live birth (defined as delivery of a fetus with signs of life at ≥28 weeks of gestation) among those that achieved clinical pregnancy. This indicator reflects the ultimate capacity to achieve a successful live birth after clinical pregnancy.
  - LBR = (number of clinical pregnancy cycles resulting in live birth/number of clinical pregnancy cycles) × 100%
  - Total miscarriage rate (MR): The proportion of cycles with spontaneous miscarriage among those that achieved clinical pregnancy. This indicator is used to evaluate the capacity for pregnancy maintenance after embryo implantation.
  - MR = (number of miscarriage cycles/number of clinical pregnancy cycles) × 100%.
  - Total ectopic pregnancy rate(EPR): The proportion of cycles diagnosed with ectopic pregnancy (gestational sac located outside the uterine cavity) among those that achieved clinical pregnancy (typically defined as the observation of at least one gestational sac on ultrasound, regardless of its location). This indicator reflects the risk of abnormal embryo implantation.
  - EPR = (number of ectopic pregnancy cycles/number of clinical pregnancy cycles) × 100%.

### 2.7 Variables

1. Demographic baseline characteristics: Age, BMI, gravidity, parity, type of infertility, and duration of infertility.
2. Reproductive variables (in the first ovarian stimulation cycle): Basal FSH (bFSH), basal LH (bLH), basal E2 (bE2), basal P (bP); duration of gonadotropin stimulation (Gn days); total dose of gonadotropins (Gn dose); trigger day LH (tLH), trigger day E2 (tE2), trigger day P (tP); and number of stimulation cycles.
3. Genetic/Embryological variables: Number of oocytes retrieved, CPR, LBR, MR and EPR.
4. Breast imaging variables: BI-RADS classification at baseline and at each follow-up examination.

If a lesion was present, its features—including size, shape, orientation, and margins—were recorded in accordance with BI-RADS criteria.

For the control group, who underwent routine health examinations, only age, BMI, gravidity, parity, and baseline and final follow-up BI-RADS classifications were recorded.

### 2.8 Data sources

1. All data were extracted from the hospital’s electronic medical record system, ensuring traceability:
2. Reproductive Medicine ART Electronic Medical Record System: Provided data on ovarian stimulation protocols, medication doses, and cycle details.
3. Picture Archiving and Communication System: All original breast ultrasound images and reports from baseline and follow-up examinations were retrieved.
4. Hospital Information System: Demographic information, reproductive history, disease history, family history, and medication history were extracted.
5. Laboratory Information System: Serum hormone levels during stimulation cycles were obtained.
6. Pathology Information System: Pathological diagnoses and immunohistochemical results of biopsy specimens were retrieved.

### 2.9 Statistical analyses

All statistical analyses were performed using SPSS software (version 27.0). First, to reduce confounding bias, PSM was performed to match the exposed group (women undergoing ovarian stimulation) with the control group (women undergoing routine health examinations) at a 1:1 ratio. Propensity scores were estimated using a multivariate logistic regression model incorporating age, BMI, gravidity, parity, and baseline BI-RADS category as covariates. The nearest neighbor matching method with a caliper width of 0.02 was applied. After matching, covariate balance was assessed using standardized mean differences (SMDs), with SMD <0.1 considered indicative of adequate balance. In the matched cohort, analyses were stratified by age (<35 years vs. ≥35 years). Continuous variables were presented as mean ± standard deviation and compared between groups using Student’s t-test (or the Mann–Whitney U test in cases of unequal variances). Categorical variables were analyzed using the chi-squared test to compare baseline characteristics and outcome measures between the two groups. Further analyses were conducted within the exposed group stratified by age. The Cochran–Armitage trend test was used to examine the linear trend between the number of stimulation cycles (1, 2, and ≥3 cycles) and the rate of BI-RADS upgrade. Odds ratios were calculated, where a larger odds ratio indicated a greater likelihood of upgrade relative to downgrade within a given subgroup. All tests were two-sided, and a P-value <0.05 was considered statistically significant.

### 2.10 Ethical approval

The study protocol was reviewed and approved by the Ethics Committee of the Affiliated Hospital of Shandong University of Traditional Chinese Medicine (Approval No. 2025-195-01-KY). Given the retrospective design of this study, which exclusively utilized existing, de-identified medical record data and posed no additional risk to patients, the requirement for informed consent was waived by the Ethics Committee. All data were handled and analyzed under strict confidentiality, in full accordance with the principles of the Declaration of Helsinki.

## 3 Results

**Figure 1:**
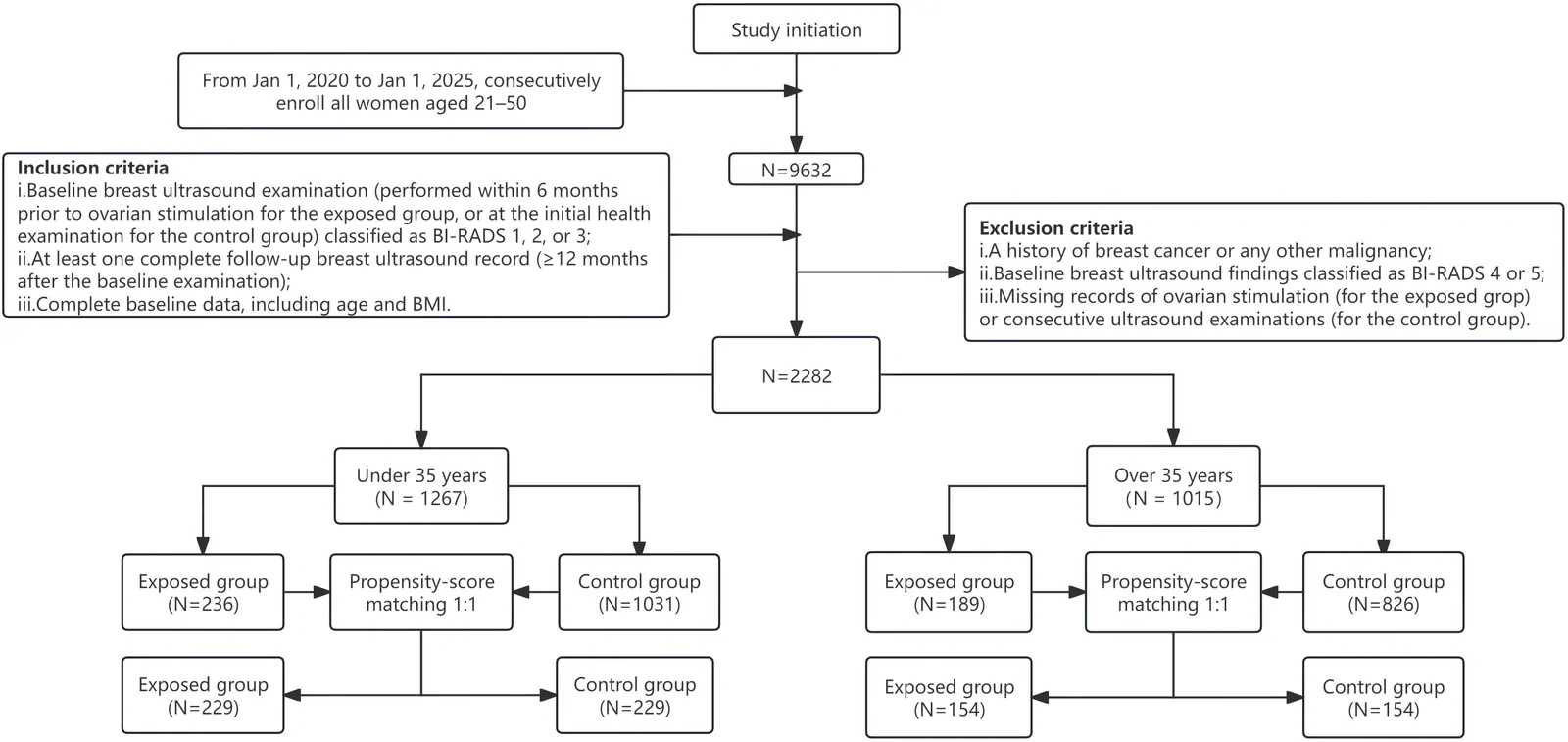
Flow diagram.

The initial study population consisted of 9, 632 women, comprising 4, 354 women who underwent COS, including 2, 922 under 35 years of age and 1, 432 aged 35 years or older, and 5, 278 healthy controls, including 3, 065 under 35 years of age and 2, 213 aged 35 years or older. Based on the predefined inclusion and exclusion criteria, a total of 2, 282 women met the criteria, and PSM was performed separately for the exposed and control groups using a cutoff age of 35 years. among women under 35 years, 229 COS-exposed women were successfully matched with an equal number of controls (N = 229). Among women aged 35 years or older, 154 COS-exposed women were successfully matched with an equal number of controls (N = 154), forming the post-matching analysis cohort (**Figure 1**). Distribution of ovarian stimulation protocols in the exposed group (**Supplementary Figures 1A, B**).

Baseline characteristics before matching showed that the SMD values for age, gravidity, and parity were all >0.1, indicating significant imbalance between the two groups on these variables (**Table 1**). After matching, the SMD values for all variables decreased to below 0.1, indicating that matching effectively eliminated intergroup differences and ensured comparability for subsequent analyses. Among women under 35 years of age, no statistically significant differences were observed between the exposed and control groups in terms of age (29.25 ± 2.91vs. 29.45 ± 2.70, P = 0.57), BMI (24.47 ± 4.45vs. 24.51 ± 4.43, P = 0.94), gravidity (0.66 ± 0.96vs. 0.66 ± 0.93, P = 0.96), parity (0.13 ± 0.37vs. 0.14 ± 0.35, P = 0.89), or duration of infertility (3.34 ± 2.04vs. 3.50 ± 1.99, P = 0.49). Similarly, good inter-group balance was demonstrated in the cohort aged 35 years or older, with no significant differences in age (38.99 ± 3.39vs. 38.87 ± 3.18, P = 0.75, BMI(24.47 ± 3.98vs. 24.65 ± 4.02, P = 0.69), gravidity (1.68 ± 1.40vs. 1.72 ± 1.38, P = 0.80), parity (0.75 ± 0.65vs. 0.73 ± 0.63, P = 0.69), or duration of infertility (4.58 ± 3.33vs. 4.62 ± 3.28, P = 0.92), indicating comparability. Follow-up duration is presented as mean (median) in months. Among women under 35 years of age, the exposed group had a mean of 14.4 and a median of 13.6, while the control group had a mean of 12.6 and a median of 12.2. Among women aged 35 years or older, the exposed group had a mean of 17.5 and a median of 16.0, while the control group had a mean of 12.3 and a median of 12.1 (**Table 2**).

**Table 1:** Baseline characteristics before PSM.

Variables	<35 years				≥35 years			
	Exposed group (N=236)	Control group (N=1031)	P-value	SMDs	Exposed group(N=187)	Control group (N=826)	P-value	SMDs
Age	29.33 ± 2.95	30.56 ± 3.08	<0.001	0.40	38.89 ± 3.41	40.11 ± 4.13	0.001	0.30
BMI	25.01 ± 4.42	25.34 ± 5.26	0.37	0.064	24.46 ± 3.97	24.92 ± 4.69	0.21	0.101
Gravidity	0.62 ± 0.95	0.74 ± 0.86	0.06	0.137	1.71 ± 1.42	1.93 ± 1.16	0.03	0.182
Parity	0.16 ± 0.32	0.39 ± 0.72	<0.001	0.346	0.66 ± 0.68	1.02 ± 0.85	0.001	0.438

**Table 2:** Baseline characteristics and overall outcome comparison between the exposed and control groups after 1:1 PSM.

Variables	<35 years					≥35 years			
	Exposed group (N=229)		Control group (N=229)	P-value	SMDs	Exposed group(N=154)	Control group (N=154)	P-value	SMDs
Age	29.25 ± 2.91		29.45 ± 2.70	0.57	0.071	38.99 ± 3.39	38.87 ± 3.18	0.75	0.030
BMI	24.47 ± 4.45		24.51 ± 4.43	0.94	0.009	24.47 ± 3.98	24.65 ± 4.02	0.69	0.037
Gravidity	0.66 ± 0.96		0.66 ± 0.93	0.96	0.005	1.68 ± 1.40	1.72 ± 1.38	0.8	0.029
Parity	0.13 ± 0.37		0.14 ± 0.35	0.89	0.013	0.75 ± 0.65	0.73 ± 0.63	0.69	0.031
Infertilit duration	3.34 ± 2.04		3.50 ± 1.99	0.49	/	4.58 ± 3.33	4.62 ± 3.28	0.92	/
Infertility type	IVF	142	/	/		86	/	/	
	ICSI	87	68						
follow-up time(month)	14.4(13.6)		12.6(12.2)	/		17.5(16.0)	12.3(12.1)	/	
Overall outcome	Elevated	69/229 (30.13%)	31/229 (13.54%)	0.019		56/154 (36.36%)	19/154 (12.33%)	<0.001	
	Reduced	34/229 (14.84%)	16/229 (6.98%)	0.007		7/154 (4.54%)	10/154 (6.49%)	0.454	

### 3.1 Primary analysis

Among women under 35 years of age, the rate of BI-RADS category upgrade was significantly higher in the COS-exposed group than in the control group (30.13% vs. 13.54%, P = 0.019), suggesting that ovarian stimulation may be associated with an increased likelihood of radiographic changes in the breast among younger women. Conversely, the rate of BI-RADS category downgrade was also higher in the exposed group compared with the control group (14.84% vs. 6.98%, P = 0.007). In the cohort aged 35 years or older, the rate of BI-RADS category upgrade remained significantly elevated in the exposed group relative to the control group (36.36% vs. 12.33%, P<0.001), corresponding to an approximately 2.9-fold higher risk of upgrade. This finding indicates that the effect of ovarian stimulation on BI-RADS category upgrade may be more pronounced in older women. However, no statistically significant difference was observed in the rate of downgrade between the two groups in this age stratum (4.54% vs. 6.49%, P = 0.454). These results suggest that, among women aged 35 years or older, ovarian stimulation is primarily associated with an increased likelihood of BI-RADS category upgrade (**Table 2**).

Among women who underwent ovarian stimulation, the rate of BI-RADS category upgrade was lower in those under 35 years of age compared with those aged 35 years or older (30.13% vs. 36.36%), suggesting that advancing age may potentiate the effect of ovarian stimulation on breast category upgrade. The rate of BI-RADS category downgrade was 14.84% in the younger group, which was higher than the 4.54% observed in the older group, indicating that younger women may exhibit greater bidirectional reversibility in breast category changes following ovarian stimulation, whereas radiographic changes in older women tend to be predominantly unidirectional progression.In the control group, the rate of BI-RADS category upgrade was similar between younger and older women (13.54% vs. 12.33%), and the rate of downgrade was also comparable (6.98% vs. 6.49%). These findings suggest that, in the absence of ovarian stimulation, BI-RADS category changes remain relatively stable across different age groups, with no evident age-related trend (**Table 2**).

### 3.2 Secondary analysis

In the first ovarian stimulation cycle of patients in the exposed group, baseline reproductive hormone levels and ovarian stimulation characteristics exhibited significant age-related differences. Women under 35 years of age had lower basal FSH levels, a significantly higher number of oocytes retrieved, and higher E2 levels on the day of trigger, along with lower total gonadotropin consumption compared with those aged 35 years or older, indicating better ovarian reserve and enhanced response to stimulation in younger women (**Table 3**).

**Table 3:** Reproductive and genetic characteristics of the first ovarian stimulation cycle in the exposed group.

Variables	<35 years(N=229)	≥35 years(N=154)
bFSH	6.65 ± 2.44	8.58 ± 6.36
bLH	5.13 ± 3.22	5.19 ± 4.26
bE2	39.11 ± 24.55	47.27 ± 30.69
bP	0.62 ± 0.38	0.63 ± 0.51
Gn duration (days)	9.55 ± 2.16	8.81 ± 3.08
Total Gn dose	2141.92 ± 817.84	2362.41 ± 1138.81
Trigger day LH	2.28 ± 1.38	3.45 ± 2.40
Trigger day E2	2967.07 ± 1791.97	1849.22 ± 1454.04
Trigger day P	1.24 ± 0.90	1.03 ± 0.72
Oocytes retrieved	13.53 ± 7.90	7.46 ± 5.94

In the analysis of the association between the number of stimulation cycles and the risk of BI-RADS category upgrade, the effect of cycle number on the risk of BI-RADS progression exhibited age-related differences. Among women under 35 years of age, the upgrade/downgrade ratio ratios for different cycle numbers remained stable, ranging from 0.46 to 0.50, with no statistically significant trend (P = 0.86). In contrast, among women aged 35 years or older, the upgrade/downgrade ratio ratios increased progressively with the number of cycles (Cycle 1: 0.10; Cycle 2: 0.15; Cycle 3: 0.33), with a significant trend (P = 0.04). These findings suggest that undergoing multiple stimulation cycles may be associated with an increased risk of BI-RADS category upgrade in older women (**Table 4**).

**Table 4:** Association between the number of stimulation cycles and risk of BI-RADS progression (subgroup analysis).

	<35 years (N = 229)					≥35 years (N = 154)				
Stimulation cycle	N	Upgrade N	Downgrade N	Upgrade/Downgrade ratio	P-value vs. cycle 1	N	Upgrade N	Downgrade N	Upgrade/Downgrade ratio	P-value vs. cycle 1
1	178	23	50	0.46	Reference	91	3	30	0.1	Reference
2	47	8	16	0.5	0.86	41	2	13	0.15	0.65
3	0	0	0	–	–	22	2	6	0.33	0.08
P fortrend²	–	–	–	–	0.86	–	–	–	–	0.04

Following ovarian stimulation, patients underwent embryo transfer. Among those under 35 years of age, the clinical pregnancy rate (37.4% vs. 28.47%) and live birth rate (62.16% vs. 33.72%) were higher than in patients aged 35 years or older, while the miscarriage rate was lower (32.43% vs. 60.46%). The ectopic pregnancy rate remained low in both groups (3.24% vs. 3.48%) (**Table 5**).

**Table 5:** Pregnancy outcomes of the exposed group.

Variables	<35 years (N = 495)	≥35 years (N = 302)
Total clinical pregnancy rate	185/495(37.37%)	86/302(28.47%)
Total live birth rate	115/185(62.16%)	29/86(33.72%)
Total miscarriage rate	60/185(32.43%)	52/86(60.46%)
Total ectopic pregnancy rate	6/185(3.24%)	3/86(3.48%)

## 4 Discussion

A key finding of this study, which tempers the concern over imaging progression, is that all 12 patients who underwent biopsy due to an upgrade to BI-RADS 4A had benign pathological results. This strongly suggests that the observed increase in BI-RADS category in the COS-exposed group predominantly reflects a benign, and potentially transient, proliferative response of breast epithelium to supraphysiological hormonal stimulation, rather than indicating a true malignant transformation. Although previous studies have largely focused on the association between ovarian stimulation and breast cancer risk (15), and large cohort studies have not confirmed a significantly increased overall risk of breast cancer (22, 23). Our study, using dynamic changes in BI-RADS as the endpoint, more sensitively captured breast imaging alterations following hormonal stimulation.

Previous international guidelines recommended that breast cancer screening for average-risk women begin at age 40–50 years (24, 25). Unlike the above studies, the present study focused on ART and used 35 years as the cutoff for advanced age in women (19, 20), a classification that follows the age stratification criteria of the POSEIDON criteria (21). This study found that among both the control and exposed groups, the risk of breast nodules in women aged ≥35 years was higher than that in women aged <35 years, which is consistent with previous studies. Age, as a key risk factor for breast cancer, has been well established in multiple studies. Evidence from breast cancer risk assessment models indicates that the risk of breast cancer in women increases significantly with age, and there are notable differences in screening intensity and cancer detection rates across different risk categories (23). From the perspective of benign breast lesions, age is also closely associated with subsequent breast cancer risk. A study involving 4, 819 patients with benign breast disease reported a median age of 51 years and found that their overall breast cancer risk was significantly higher than that of the general population (SIR = 1.95; 95% CI, 1.76–2.17); Additionally, it was suggested that the risk level increases with the severity of pathological findings and the number of lesions, indicating that breast nodules themselves, along with their interaction with age, may jointly contribute to the development of breast cancer (26). Furthermore, a systematic review encompassing 67 studies also noted that among women with biopsy-confirmed benign breast disease, older age was associated with a higher subsequent risk of breast cancer (27). In the present study, compared with women under 35 years of age, those aged 35 years or older not only had a higher rate of progression but also exhibited a greater disparity between the exposed and control groups, suggesting that age may amplify the imaging progression effect of ovarian stimulation by increasing the sensitivity of breast epithelium to hormonal stimulation.

In addition to comparing the rate of breast nodule progression between the two groups, we also analyzed the rate of regression. The results showed that in women aged <35 years, the exposed group not only had a higher incidence of BI-RADS progression or new nodule formation than the control group, but also exhibited a significantly higher rate of regression during follow-up. Therefore, we tentatively propose an exploratory hypothesis of ‘pregnancy-lactation remodeling’ as a potential biological explanation for the observed higher regression rate in younger women. However, it must be strongly emphasized that this hypothesis remains speculative. Several studies have suggested that postpartum breastfeeding may influence breast biology through multiple mechanisms. For instance, lactation may exert anti-tumor effects in some women by activating pro-apoptotic pathways associated with endoplasmic reticulum stress (28); Furthermore, studies have shown that breastfeeding can induce the accumulation of CD8+ T cells in breast tissue, enhance immune surveillance, and improve prognosis in patients with triple-negative breast cancer (29). More notably, a complete pregnancy–lactation cycle can induce terminal differentiation of mammary epithelial cells, conferring long-term protective effects against breast cancer, particularly against hormone receptor-positive (HR+) subtypes (30–32). During this period, the breast undergoes a series of highly ordered physiological changes, including development, secretion, and involution, which help eliminate undifferentiated cells with DNA damage. Prolactin promotes functional maturation of the mammary epithelium and inhibits aberrant proliferation by activating the JAK2/STAT5/mTOR signaling pathway (33); moreover, normal programmed involution following lactation reduces chronic inflammation and the formation of a pro-tumorigenic microenvironment (30, 34). Therefore, we propose an exploratory hypothesis of ‘pregnancy-lactation remodeling’: the processes of pregnancy and lactation following ovarian stimulation may provide an opportunity for structural and functional remodeling of breast tissue, thereby counteracting prior proliferative stimulation and promoting nodule regression. However, it must be acknowledged that the present study did not directly collect data on patients’ breastfeeding behaviors (e.g., duration of breastfeeding, feeding method, etc.), so this hypothesis still requires validation through prospective studies combined with detailed breastfeeding history follow-up. Nevertheless, we have extensively reviewed the literature exploring the association between breastfeeding and the risk of breast disease. For example, a collaborative analysis integrating 47 epidemiological studies clearly indicated that longer duration of breastfeeding is positively associated with a reduced risk of breast cancer (35), meaning that more prolonged breastfeeding may lower the risk of breast cancer (36, 37). Additionally, in women aged ≥35 years, the regression rate in the exposed group was slightly lower than that in the control group. We speculate that among women in this age group, some breast nodules may be at the borderline of precancerous lesions or exhibit a higher tendency for malignant transformation. Such nodules are more likely to progress rather than regress in response to hormonal stimulation, thereby reducing the overall regression rate.

Although the rate of BI-RADS progression was significantly higher in the exposed group in this study, it should be noted that all 12 patients who underwent biopsy due to upgrading to BI-RADS 4A during follow-up had benign pathological findings. This finding provides important clinical reassurance: most of the imaging progression induced by ovarian stimulation may reflect benign, possibly transient hormonal effects rather than true malignant progression. Therefore, is not to label patients as being at ‘high risk’ for cancer, but rather to implement a structured, yet reassuring, imaging follow-up protocol. An upgrade to BI-RADS 4A in this context should prompt a biopsy for definitive diagnosis, which, as our data show, is very likely to be benign. This approach avoids unnecessary anxiety and overtreatment while ensuring that any true malignancies are not missed.

The difference in follow-up duration between the exposed and control groups arises from distinct clinical pathways rather than from a study design flaw. Theoretically, this discrepancy may introduce detection-time bias, whereby a longer observation window increases the opportunity to capture dynamic changes in BI-RADS categories. However, our data analysis indicates that if prolonged follow-up were the sole driver, both upgrade and downgrade rates should have increased concurrently. In women aged ≥35 years, despite having the longest follow-up, the exposed group showed a marked increase in upgrade rates but no increase in downgrade rates. This discordant pattern argues against pure time-related bias and instead reflects a genuine biological effect of COS, which appears to be magnified by age. In women under 35 years, the exposed group exhibited a significantly higher downgrade rate, which we exploratorily attribute to nodule regression induced by post-pregnancy lactation—a biological process rather than a bias.

First, methodologically, this study employed PSM and blind image re-review, ensuring the reliability of the results. Potential confounding factors were effectively controlled, and baseline balance between the exposed and control groups was achieved. Second, the study focused on the dynamic imaging changes in the breast following ovarian stimulation rather than on traditional malignant endpoints. Previous studies have largely concentrated on the epidemiological association between ART and breast cancer risk or on short-term effects of hormones on single imaging parameters such as breast density. In contrast, this study is the first to use BI-RADS classification as a dynamic observational indicator to systematically evaluate the imaging impact of COS on breast nodule progression and regression. As a core tool for clinical decision-making, changes in BI-RADS classification may more sensitively reflect early breast tissue responses to hormonal stimulation, offering a novel perspective and intermediate phenotype for assessing ART-related breast risks. Furthermore, through age-stratified analysis, the modifying effect of age on the breast response to ovarian stimulation was elucidated. This finding provides important evidence for developing individualized breast monitoring strategies for women of different ages in clinical practice. Finally, this study revealed the bidirectional characteristics of breast nodule changes following ovarian stimulation and, for the first time, proposes an exploratory hypothesis—the “pregnancy–lactation remodeling hypothesis” —to explain the observed nodule regression in young women. This finding offers a new biological perspective for understanding the complex relationship between hormonal exposure and breast tissue plasticity.

Although this study was designed and analyzed with rigor, several limitations should be acknowledged. First, due to the retrospective design of this study, the causes of infertility and first-degree family history of breast cancer were not systematically recorded. Consequently, these two key covariates could not be included in the PSM, and this omission may introduce potential confounding bias, representing a primary limitation of this study. Moreover, the choice of controls from a general health screening population could introduce indication bias, because these women might have different reproductive histories, healthcare-seeking patterns, and baseline endocrine profiles compared with ART patients. Despite PSM minimizing observable imbalances, unmeasured confounding cannot be fully ruled out. Furthermore, the follow-up time point in this study was set at ≥12 months after the baseline examination, but critical time-dependent variables such as postpartum breastfeeding status and breastfeeding duration were not collected. Therefore, the current analysis cannot adequately assess the long-term effects of ovarian stimulation on breast nodules. Consequently, our proposed ‘pregnancy-lactation remodeling’ hypothesis should be regarded as a tentative speculation rather than a definitive conclusion. Future prospective studies incorporating these time-dependent variables and extending follow-up duration are warranted to more accurately evaluate the causal relationships.

In summary, this study demonstrates that ovarian stimulation in ART is associated with short-term, imaging-detectable changes in BI-RADS categories, particularly in women aged ≥35 years. Crucially, these imaging changes, when biopsied, proved to be uniformly benign. The clinical implication of this finding is not to suggest an increased risk of breast cancer, but to highlight the importance of structured imaging surveillance during and after ART. The higher regression rate observed in younger women in the exposed group, which we speculatively attribute to potential subsequent pregnancy and lactation, underscores the dynamic nature of these imaging changes. However, in the absence of direct breastfeeding data, this interpretation remains exploratory and requires confirmation in future studies. Future prospective studies with extended follow-up, detailed reproductive histories, and of breastfeeding status and duration are necessary to validate our primary findings and to rigorously test the speculative ‘pregnancy-lactation remodeling’ hypothesis.

## Data Availability

The raw data supporting the conclusions of this article will be made available by the authors, without undue reservation.
