# Supplementary material for: Does ovarian stimulation accelerate the progression of BI-RADS categories? a propensity score matching-based retrospective cohort study
Source: Front Endocrinol (Lausanne). 2026 Aug 11;17:1844265. doi: 10.3389/fendo.2026.1844265 (PMC13503318; doi:10.3389/fendo.2026.1844265)
Supplement: Supplementary file 1 [file DataSheet1.docx]

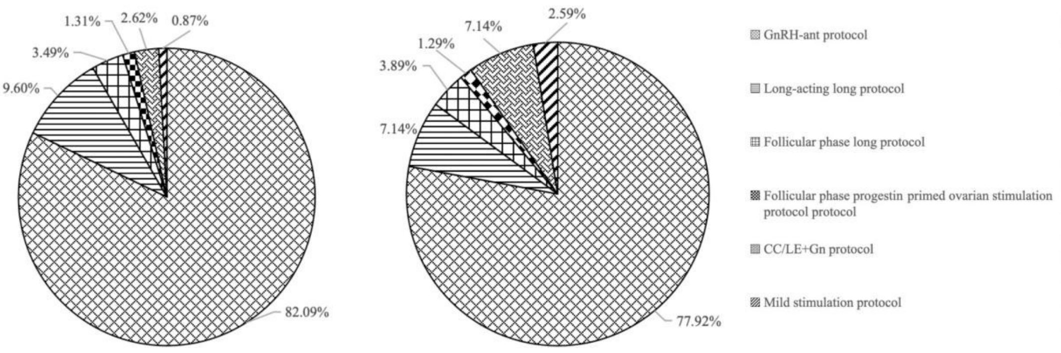

1.31%
2.62%
0.87%
3.49%
9.60%
82.09%
7.14%
2.59%
1.29%
3.89%
7.14%
77.92%
GnRH-ant protocol
Long-acting long protocol
Follicular phase long protocol
Follicular phase progestin primed ovarian stimulation protocol protocol
CC/LE+Gn protocol
Mild stimulation protocol
